# Island Syndrome in the Critically Endangered Lord Howe Island Cockroach *Panesthia lata*

**DOI:** 10.1002/ece3.72402

**Published:** 2025-11-04

**Authors:** Susannah K. Coady, Maxim W. D. Adams, Harley A. Rose, James A. Walker, Ian Hutton, Ros Gloag, Nathan Lo

**Affiliations:** ^1^ School of Life and Environmental Sciences University of Sydney Sydney New South Wales Australia; ^2^ Australian Government Department of Agriculture, Water and Environment Canberra Australian Capital Territory Australia; ^3^ Lord Howe Island Museum Lord Howe Island New South Wales Australia

**Keywords:** animal behaviour, conservation, insects, island biology, island syndrome

## Abstract

Following island colonisation, organisms experience a unique array of selective pressures, giving rise to a somewhat predictable suite of morphological, demographic and ecological adaptations known as the “island syndrome”. Studies of the island syndrome have provided valuable insights into processes of speciation, community assembly, adaptive radiation and ecological release, alongside many others. However, to date, behavioural aspects of island adaptation have comparatively received little scientific attention, especially among invertebrates. In this study, we examined the agonistic, courtship and aggregation behaviour of the critically endangered Lord Howe Island cockroach, *Panesthia lata*, and compared these to its Australian sister species, *Panesthia cribrata*. Behavioural assays revealed that while courtship behaviour was relatively stable across the two species, there was a significantly lower incidence of male agonism in *P. lata* . In concordance, analyses of nuclear single‐nucleotide polymorphisms showed that *P. lata* forms large aggregations of unrelated individuals, unlike most *Panesthia* species, which maintain stable family groups. These results align with previous findings of relaxed intraspecific aggression in island mammals and reptiles, providing novel evidence of behavioural island syndrome in an invertebrate. We also found that courtship behaviour did not vary when *P. lata* interacted with conspecifics from the same or different populations, suggesting that individuals from different populations will readily interbreed. This is a promising outcome for the conservation of this critically endangered species, which currently spans a fragmentary range consisting of small, insular populations.

## Graphical abstract

The Lord Howe Island cockroach Panesthia lata was found to display significantly reduced intraspecific agonism, and to aggregate more readily with non‐kin, than its mainland sister species Panesthia cribrata. This represents the first evidence that an invertebrate species undergoes similar behavioural shifts as vertebrates following island colonisation.

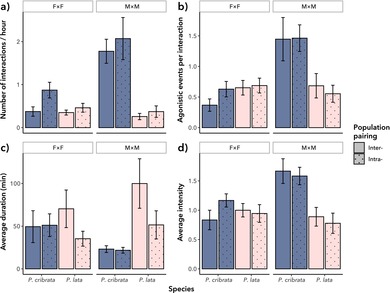

## 1 Introduction

Islands are valuable natural laboratories in which to study the process of evolution. Characteristics such as insularity, environmental unpredictability, and low species diversity foster unique selective pressures (Covas 2012; Losos and Ricklefs 2009). As a result, many island taxa differ morphologically, ecologically, physiologically, and demographically from mainland relatives, a phenomenon collectively termed the “island syndrome” (Baeckens and Van Damme 2020). While the generality of island syndrome remains highly contested (reviewed by Jezierski et al. 2024), a number of adaptations appear to arise convergently across a broad swathe of taxa. For example, selection against flight has led to wing reduction in many insular birds and invertebrates (Leihy and Chown 2020; Wright et al. 2016). Other widely reported adaptations include reduced reproductive output, higher life expectancy, heightened sexual dimorphism and potentially changes in body size (Adler and Levins 1994; Baeckens and Van Damme 2020; Benítez‐López et al. 2021; Sen et al. 2014; Whittaker et al. 2023), but see (Biddick and Burns 2021; Meiri et al. 2004, 2006).

Despite the adaptive significance of behaviour, there have been comparatively few examinations of the behavioural components of island syndrome. Island species often experience increased population density and ecological release, the combination of which appears to reduce boldness, aggression, and intra‐specific agonism (Baeckens and Van Damme 2020). Similarly, the trend towards reduced fecundity may co‐evolve with shifts in mating and parental care strategies (Covas 2012). However, few studies have experimentally compared island species with mainland species, in particular for more complex traits such as behavioural syndromes (reviewed by Gavriilidi et al. 2022). Moreover, despite the fact that invertebrates are relatively over‐represented in island ecosystems, research on island behaviour has overwhelmingly focused on vertebrates (reviewed by Gavriilidi et al. 2022; Meiri 2007; New 2008; Whittaker et al. 2023).

**FIGURE 1:**
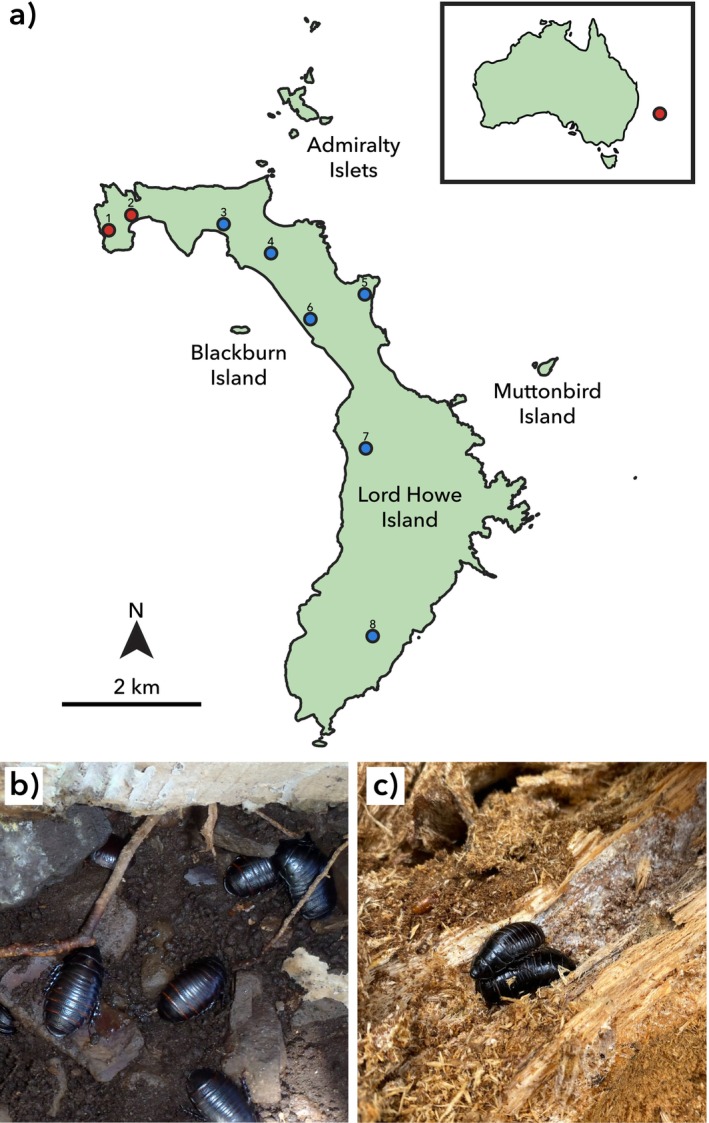
(a) Map of the Lord Howe Island Group. Red dots denote the locations of the (1) North Head and (2) North Bay populations of *Panesthia lata*. Blue dots denote the locations where surveys were conducted without detection of *Panesthia lata*: (3) Old Settlement, (4) Stevens Reserve, (5) Valley of Shadows, (6) Lagoon Road, (7) Soldiers Creek, and (8) Mount Gower. Inset: position of the islands relative to mainland Australia. (b) Large aggregation of *Panesthia lata* in soil on Blackburn Island. Photo: N. Carlile. (c) Aggregation of *Panesthia cribrata* within a *Melaleuca* sp. log in Bournda, New South Wales. Photo: M.W.D. Adams.

One potentially informative system in which to investigate the behaviour of island invertebrates is the Lord Howe Island Group (LHIG) of Australia. Located *ca*. 780 km northeast of Sydney, the LHIG comprises 28 islets and rocks, including Lord Howe Island (LHI) itself, with a total area of only 15 km^2^ (Green 1993; Figure 1a). The islands support a diverse and highly endemic biota, which is overwhelmingly dominated by invertebrates (DECC 2007). Surveys have reported over 1600 species of terrestrial invertebrates alone, with many more yet to be described (Cassis et al. 2003). While biogeographic studies are scarce, species appear to originate mainly from Australia, with contributions from New Zealand, New Caledonia, and other Pacific islands (Green 1993). Small and remote islands such as the LHIG are thought to select most strongly for island syndrome (Whittaker and Fernández‐Palacios 2007); thus, a comparison of an LHIG‐endemic invertebrate with Australian relatives has great potential to reveal behavioural adaptations to island life.

The Lord Howe Island cockroach *Panesthia lata* is a large, flightless insect endemic to the LHIG, estimated to have reached the islands *ca*. 2 Ma (Adams, Walker, Rose, Jones, et al. 2025). The species shows multiple ecological shifts relative to its congeners on the Australian mainland. The majority of *Panesthia* species reside within and feed upon decaying logs, aggregating in family groups that consist of one or both parents and several offspring. In the sister species of *P. lata* , *Panesthia cribrata sensu stricto* ( *P. cribrata* South; Adams, Walker, Rose, Jones, et al. 2025), females likely remain within a single log for the duration of their lives, whereas males disperse at maturity (Rugg and Rose 1984). In contrast, *P. lata* resides in shallow soil burrows beneath stones or logs, forming larger aggregations comprising multiple generations of adults and/or nymphs (up to *ca*. 15 individuals; M.W.D. Adams, pers. obs.). Individuals are also thought to leave the resident stone at night to forage for woody material and leaves (Carlile et al. 2018). While male *P. cribrata* readily engage in agonistic behaviours (O'Neill et al. 1987), it is unknown whether the higher aggregation densities of *P. lata* entail a reduction of intraspecific aggression, as is found in many island vertebrates. In addition, kinship within and between its nesting aggregations has never been investigated with genetic data.

Behavioural study of *P. lata* will not only illuminate behavioural adaptations to island life, but also augment a knowledge base dedicated to the species' conservation. *Panesthia lata* is present on multiple islands of the LHIG, with the populations on Blackburn Island, the Admiralty Islets, and LHI having been isolated by sea‐level inundation for at least 10,000 years (Adams, Ewart, Carlile, Rose, et al. 2025; Figure 1a). It was thought that the population on LHI was locally extirpated by invasive black rats ( *Rattus rattus* ), leading the species to be listed as Endangered by the NSW Threatened Species Scientific Committee (2004), followed by a recent reassessment as Critically Endangered (NSW Threatened Species Scientific Committee 2025). However, following the successful eradication of rodents in 2019, a relict population was discovered in LHI's North Bay. The authors have shown that this population, and those on the smaller islands, are highly inbred, which may elevate the species' extinction risk (Adams, Ewart, Carlile, Rose, et al. 2025). Further knowledge of the ecology and behaviour of the different populations will enhance future conservation actions.

In this study, we undertake further surveys of LHI and examine the behaviour of *P. lata* . By examining social, agonistic, and courtship behaviours in multiple populations across the LHIG and comparing these to *P. cribrata* , we aim to: (1) determine whether *P. lata* displays behavioural changes consistent with the island syndrome; and (2) test whether courtship and agonistic behaviours vary between populations of *P. lata* . We also analyse a panel of nuclear single‐nucleotide polymorphisms (SNPs) to (3) characterise patterns of genetic relatedness within nesting aggregations of *P. lata* . Our results reveal complex behavioural shifts following island colonisation and provide further information to guide the management of this critically endangered species.

## 2 Materials and Methods

### 2.1 Habitat Surveys and Specimen Collection

Over four separate expeditions between 2022 and 2025, we opportunistically examined suitable habitats across LHI for the presence of *P. lata* . Informed by the species' habitat preferences on Blackburn Island (Carlile et al. 2018; Rose 2003), we focused our search on sites with moist soil, dense canopy and abundant ground cover consisting of decaying logs and/or stones, with a particular emphasis on areas in the vicinity of Banyan trees ( *Ficus macrophylla* var. columnaris). In total, we surveyed six different regions of the island: Old Settlement, Stevens Reserve, Valley of Shadows, Soldiers Creek, along parts of Lagoon Road, and along the track leading to the summit of Mount Gower (Figure 1a).

At a given location, we searched for cockroaches by overturning leaf litter and any large (> *ca*. 20 cm length) stones, as well as breaking any large (> *ca*. 10 cm diameter) logs. All surveys were undertaken during the day. No cockroaches or traces were found at all but two sites. In addition to a previously discovered relict population in North Bay (Adams, Ewart, Carlile, Rose, et al. 2025; Figure 1), individuals of *P. lata* were detected by Toby Kovacs, S. Coady, and N. Lo for the first time at a site on the island's North Head (Figure 1a) on May 24th, 2023. At this location, both adult and juvenile cockroaches were found sheltering beneath stones directly under the canopy of a single *F. macrophylla* tree. Despite its geographic proximity to North Bay (*ca*. 400 m), we consider this a separate population due to the lack of suitable intervening habitat. Further ecological observations, based on a transect conducted on February 20th, 2025, are outlined in the Supporting Information.

In May 2023, individuals of *P. lata* were collected from three separate populations for our behavioural assays: Blackburn Island (31.534° S 159.060° E), North Bay, LHI (31.517° S 159.042° E), and North Head, LHI (31.517° S 159.038° E). Before collection, we surveyed both relict populations to ensure sufficient population density. Cockroaches were collected in “aggregations”, which we defined as all individuals under a single rock or log. We collected six aggregations from Blackburn Island, nine from North Bay and six from North Head.

We collected specimens of *P. cribrata* from two separate localities in New South Wales: Harold Reid Reserve, Sydney (33.796° S 151.212° E) and Monga State Conservation Area, Braidwood (35.445° S 149.900° E). Collections were undertaken between April and August 2023. We collected discrete aggregations as follows: decaying logs, branches or tree trunks were overturned, and any visible cockroaches were collected; logs were then prised open to search for resident galleries containing additional cockroaches. Individuals in connected galleries within the same log, or in soil < 2 m from the galleries, were considered a single aggregation, while individuals in unconnected galleries or separated by > 2 m were considered a separate aggregation. In total, five aggregations were collected from Sydney, and nine were collected from Braidwood. Cockroaches from both species were cultured at ambient temperature in their aggregation groups and provided with coconut coir as substrate. Water and wood chips were provided *ad libitum*. To permit identification in behavioural assays, each adult was marked with a uniquely coloured and numbered acrylic marker indicating its population and aggregation, which was glued onto the metanotum.

Individuals were initially identified to species based on collection locality, as informed by a recent phylogenetic study of the Australian *Panesthia* (Adams, Walker, Rose, Jones, et al. 2025). Identifications were then confirmed via morphological examination, following the taxonomic keys of Roth (1977, 1979) and Adams, Walker, Rose, and Lo (2025). Briefly, *P. lata* was identified based on the combination of highly reduced tegmina, absent hindwings, sub‐rectangular cerci, and the entire hind margin of the supra‐anal plate; *P. cribrata* was identified based on the presence of tegmina and hindwings, dorsally glabrous cerci, and the crenulate hind margin of the supra‐anal plate.

Finally, to investigate pairwise kinship within and between nesting aggregations of *P. lata* , we collected samples for SNP data generation in July 2022. We collected individuals from under four separate rocks on Blackburn Island, chosen such that each rock was more than 5 m from all others in the sample (comfortably exceeding the expected foraging range of *P. lata* in a single night, ≤ 1.12 m; Carlile et al. 2018). Care was taken to collect every individual present under each rock, and samples were stored in 96%–100% ethanol before DNA extraction. The number of individuals in each aggregation ranged between *n* = 3 and 9. In addition, we retrieved putative unrelated individuals (i.e., all from separate aggregations) from Blackburn Island, Roach Island, and North Bay, LHI, to include as baseline reference points in our analyses. Details of their collection can be found in Adams, Ewart, Carlile, Rose, et al. (2025).

### 2.2 Aggregation Size, Morphometrics and Nesting Behaviour

We recorded the total number of individuals in each collected aggregation and measured the body length of all available adults of *P. lata* and *P. cribrata* . Live measurements were taken with a ruler to the nearest millimetre, measured from the anteromedial margin of the pronotum to the posteromedial margin of the supraanal plate.

We tested whether aggregation size varied across all five populations (from both species) using a Kruskal‐Wallis test in R. We also used a Kruskal‐Wallis test to determine whether body length differed between populations, performed separately for males and females. Where significant overall differences were found, we performed post hoc individual comparisons using Dunn tests (package *dunn.test* v.1.3.6; Dinno 2017) with Bonferroni corrections.

Carlile et al. (2018) previously conducted ecological observations of *P. lata* , suggesting that the species is primarily nocturnal and may travel small distances (≤ *ca*. 1 m) at night. However, these findings were based on observations of few individuals, and Carlile et al. (2018) emphasised the need for a follow‐up study. To investigate nesting behaviour and movement by *P. lata* , we first performed two marking experiments on Blackburn Island, on May 19th 2023 and September 14th 2024. In each case, all individuals under each of 6 rocks, spaced > 5 m from each other under the single *Ficus* tree on the island, were marked with a different colour of nail polish across their metanotum and dorsal abdomens (an area of *ca*. 3 cm^2^ in adults). The rocks under which they were found were marked, and individuals were returned to their respective rocks. On the evenings of May 23rd and September 17th, the six marked rocks, as well as rocks within 1–2 m of the marked rocks, were checked for the presence of *P. lata* , with each individual carefully checked for the presence of nail polish.

Second, we conducted observations of *P. lata* under Blackburn Island's *Ficus* tree at 9 pm on May 23rd, 2023. This was to further confirm that the species is nocturnal and whether it forages in the open or remains under leaf litter. Initial general observations were made prior to conducting a survey where three field workers walked from one side of the *Ficus* to the other (*ca*. 20 m), recording the life stage (adult or nymph) and sex of any *P. lata* seen on the ground (*ca*. 20 min of observation).

### 2.3 Behavioural Assays

**FIGURE 2:**
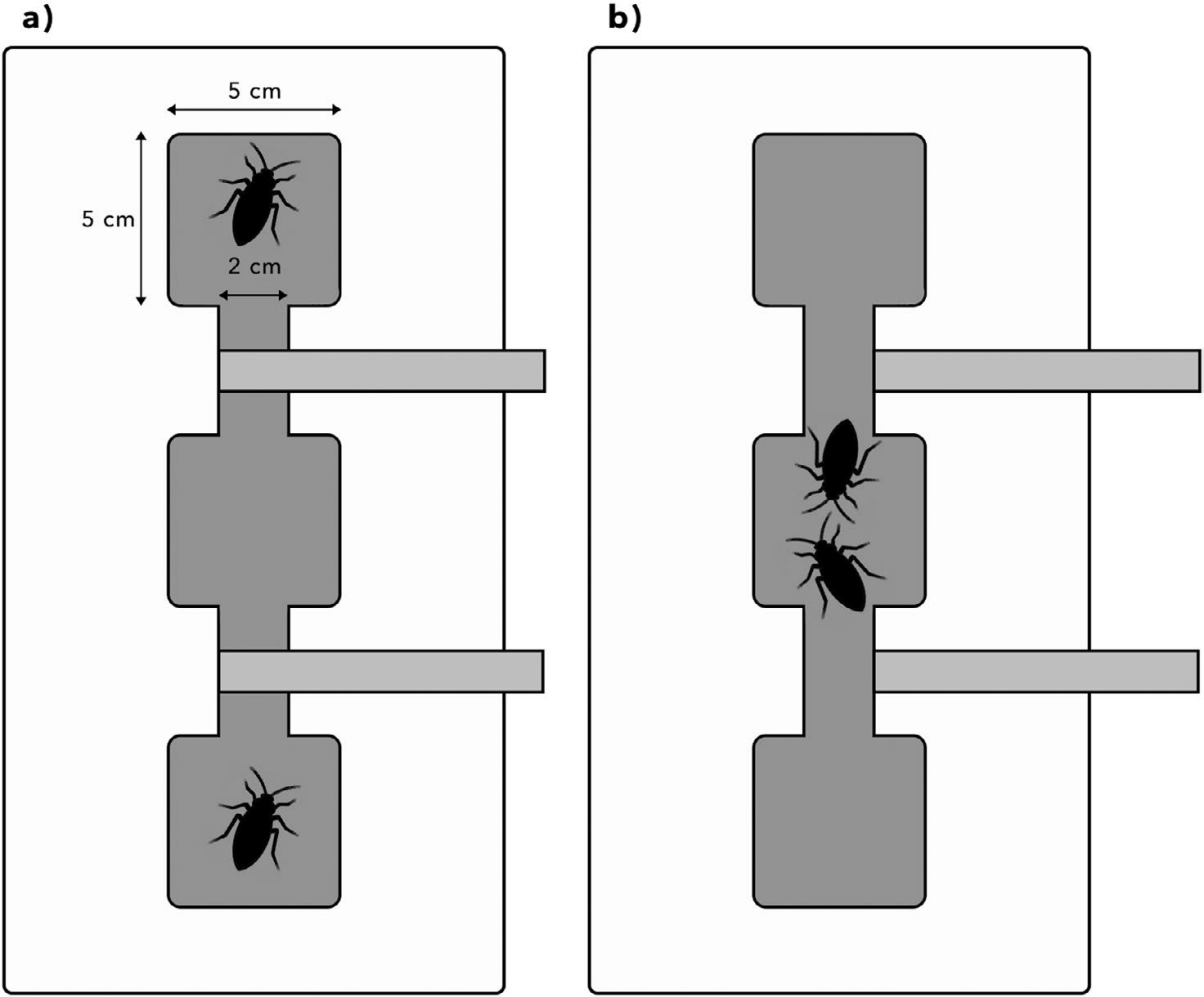
Top‐down schematic diagram of a behavioural arena. Dark shaded region represents three chambers with intervening corridors. Light shaded rectangles represent wooden rods, which are slotted into carved grooves and can be moved left or right (in top‐down view). (a) Prior to the behavioural assay, wooden barriers prevent interaction. (b) Barriers have been withdrawn to initiate the assay and permit interaction between cockroaches.

We examined behaviour using a custom‐built arena. This consisted of 3 *ca*. 5 × 5 cm chambers, connected by corridors *ca*. 2 cm wide (Figure 2), carved into a pine‐wood block to a uniform depth of *ca*. 1.5 cm. The arena was covered by a sheet of translucent Perspex to maintain humidity. Chambers were furnished with peat moss, as well as small pieces of wood or *Ficus* leaves for *P. cribrata* and *P. lata* , respectively. Behavioural experiments were conducted in September 2023 to coincide with the maturation of female oocytes and initiation of reproductive receptivity in *Panesthia*, which appear to occur in early Spring (Ito and Osawa 2025; Rugg and Rose 1989).

We investigated agonism and courtship by conducting behavioural assays with pairs of adult cockroaches. In each assay, two marked cockroaches were placed in opposite chambers of the arena, initially with retractable wooden barriers blocking the corridors to prevent their exploration (Figure 2a). These were left undisturbed in a darkened cabinet for 12 h to acclimate. To begin the assay, the barriers were manually withdrawn (Figure 2b) and the cockroaches' interactions were recorded for *ca*. 8 h using an iPhone camera. As *Panesthia* species are nocturnal and photophobic (O'Neill et al. 1987), each assay was initiated at approximately 10 pm, and the arena was illuminated using red LED lighting. Agonistic assays consisted of male–male or female–female pairings, while courtship assays were conducted using male–female pairs (Table 1).

**TABLE 1:** Nine treatment categories used to assess agonism and courtship behaviours in *P. lata* (three populations: BI = Blackburn Island, NB = North Bay, NH = North Head) and *P. cribrata* (two populations: SYD = Sydney, Brd = Braidwood). Six replicates were performed for each category. Treatments assessed the behaviours of *Panethsia* when interacting with members of their own populations, or conspecifics from different populations, and when interacting with either members of the same or different sexes.

	*P. lata*						*P. cribrata*		
	Same			Different			Same		Different
	NB × NB	NH × NH	BI × BI	NB × NH	NB × BI	NH × BI	Syd × Syd	Brd × Brd	Syd × Brd
Agonism									
M × M	6	6	6	6	6	6	6	6	6
F × F	6	6	6	6	6	6	6	6	6
Courtship									
M × F	6	6	6	6	6	6	6	6	6

Agonistic and courtship assays were conducted separately for *P. lata* and *P. cribrata* . For each species, we used a fully crossed design, whereby we tested behaviour using pairs of cockroaches from within each population and each combination thereof (Table 1). For intra‐population assays, the two cockroaches were taken from different aggregations. We conducted six replicates of each of the nine population pairings, for all three sex pairings (male–male, female–female and male–female).

Videos were viewed and scored using QuickTime Player v.10.5 (Apple Computer, USA). Primarily following O'Neill et al. (1987), we recognised 13 different behaviours across three behavioural categories: neutral, agonistic, or courtship (Table 2). For each video, we recorded the number and types of behaviours, the number of discrete interactions, the duration of each interaction, and the behavioural category of each interaction (neutral, agonistic, or courtship). We considered an interaction to have begun when both cockroaches had > 50% of their body length within the same or adjacent chamber or corridor. We considered the interaction to have ended once the cockroaches had > 50% of their body length in separate chambers for over 60 s. The behavioural category of each interaction was assigned based on the modal behaviour.

**TABLE 2:** Categories of *Panesthia* behaviours (following O'Neill et al. 1987).

Behaviour	Definition
Neutral	
Mutual antennation	Individuals investigate each other through antennae to antennae contact.
Antennation	Male or female cockroach touches the other cockroach with its antennae.
Tapping	Male touches the body of the female with his maxillary and/or labial palps. In some instances, the male also placed 1 or more of his legs on the dorsal surface of the female whilst touching the female with his palps.
Agonistic	
Pushing	Cockroaches lower their pronota by pulling their heads down towards their legs. In this posture the opponents push into each other with their pronota.
Pulsing	The cockroach extends and contracts its abdomen anteroposteriorly.
Blocking	The cockroach turns away from its opponent and lowers the side of the body or the tip of the abdomen facing its opponent. An individual could only block effectively when located in a small gallery where it could thus jam its body in the gap and prevent the other cockroach from moving or getting past it. Blocking was interpreted primarily as a defensive act.
Submission	The cockroach stands still, abdomen downturned at the tip, and antennae motionless.
Courtship	
Waggling	Male moves his abdomen rapidly back and forth in a sideways motion.
Tapping and waggling	Male taps and waggles simultaneously.
Pushing (courtship)	Female or male pushes the other with the pronotum; these pushes not as vigorous or sustained as in aggressive behaviour.
Bending	Male bends his body sideways into a “c” shape, orienting his posterior towards that of the female.
Copulation	Mating occurs
Rubbing*	Male repeatedly rubs the posterior end of his body on the female

* a newly defined behaviour.

We quantified behaviour using four outcomes: the number of agonistic/courtship interactions per hour, the proportion of agonistic/courtship interactions from the total number of interactions, the average duration of agonistic/courtship interactions, and the intensity of agonistic/courtship interactions. Intensity was scored as the number of different behaviours per interaction. After confirming data normality and homoscedasticity with Shapiro–Wilk and Levene's tests, respectively, we used generalised linear models in R to test whether these outcomes varied between species or between population pairings (whether the pairing comprised cockroaches of the same or different populations), and to check for interactions between these factors.

Second, for both agonistic and courtship interactions, we quantified the number of times each specific behaviour was performed per interaction (Table 1). We then tested whether the frequencies and types of behaviour varied between species and population pairings using a two‐way multivariate analysis of variance (MANOVA) in R. Where significant differences or interactions were found, we undertook post hoc tests examining variations in individual behaviours using two‐way ANOVAs with Bonferroni corrections.

### 2.4 SNP Data Generation and Analysis

We undertook genetic analyses using the four nesting aggregations of *P. lata* collected on Blackburn Island, as well as the putative unrelated individuals from Blackburn Island, Roach Island, and North Bay (*n* = 67 individuals in total). No North Head samples were included in genetic analyses as the population was discovered after DNA sequencing. For the majority of samples, DNA extractions were carried out in‐house, using a DNeasy Blood and Tissue Kit (Qiagen, Germany) per manufacturer's instructions. We checked DNA purity and concentration using a NanoDrop 2000 spectrophotometer (Thermo Fisher Scientific, USA) and a Qubit 2.0 fluorometer (Invitrogen, USA), respectively. For a minority of samples, DNA extraction was instead outsourced to the Australian National Insect Collection, Canberra (see Adams, Walker, Rose, Jones, et al. 2025 for details; Table S1).

Reduced‐representation sequencing and SNP data generation were carried out by Diversity Arrays Technology (DArT), Canberra. A subset of the data was previously analysed by Adams, Ewart, Carlile, et al. (2025), and sequencing methods are described in detail therein. In summary, DNA was digested with the *PstI* and *HpaII* restriction enzymes, sequenced using a NovaSeq 6000 S1, and processed through the proprietary DArT analytical pipeline.

Data were filtered chiefly using the R package *dartR* v.2.7.2 (Gruber et al. 2018). We first filtered for data quality and completeness, based on reproducibility (100%), minor allele frequency (MAF > 0.025; based on 3/2 *N*) and call rate (> 0.8, calculated for both individuals and loci). This “quality‐filtered” dataset comprised 11,446 SNPs from 62 individuals. We then produced a more stringently filtered dataset of putatively unlinked and neutral SNPs. Monomorphic loci were removed, and we retained one randomly selected SNP per sequence tag (removed “secondaries”). We also removed loci out of Hardy–Weinberg equilibrium based on the exact test (alpha value = 0.01), calculated with the mid‐p method. Finally, we confirmed the absence of any loci potentially under selection (based on elevated *F*_ST_ values) using BayeScan v.2.1 (Foll and Gaggiotti 2008) under default settings. The “neutral” dataset comprised 8006 SNPs from 62 individuals. Average coverage for both data sets was *ca*. 12 ×.

We used two approaches to explore the relatedness of individuals within and between the Blackburn Island aggregations. First, we conducted kinship analyses using the quality‐filtered SNP panel, with individuals from Blackburn Island, Roach Island, and North Bay included as putative unrelated reference points. We identified any closely related pairs (parents, grandparents, or siblings) using the R package *Sequoia* v.2.11.2 (Huisman 2017). In addition, we calculated individual pairwise kinship coefficients using maximum likelihood in the R package *SNPRelate* v.1.33.0 (Zheng 2013). These values were averaged within each nesting aggregation (excluding self‐kinship), as well as within the remaining independent individuals from Blackburn Island.

Second, we visualised genetic structure within and between nesting aggregations. We pruned the dataset to include only the four Blackburn Island aggregations and re‐filtered the raw SNPs as above (producing a quality‐filtered panel of 8926 SNPs and a neutral panel of 6471 SNPs, both from 24 individuals). Using the quality‐filtered SNPs, we conducted a principal coordinates analysis (PCoA) of individual allele frequencies in *dartR*. Using the neutral SNPs, we analysed population structure using a sparse non‐negative matrix factorisation algorithm (sNMF) in the R package *LEA* v.2.0 (Frichot and François 2015). We searched for the optimum number of genetic clusters between *K* = 1 and *K* = 8, with 10 replicates for each *K* value.

## 3 Results

### 3.1 Nesting Ecology of *Panesthia lata*

In 2023, 33 wild individuals were marked with nail polish on Blackburn Island, across 6 separate aggregations. When the location was re‐surveyed after four days, only a single marked individual was re‐captured, found under a separate rock *ca*. 20 cm from the site of its first capture. Based on pilot studies, we cannot exclude the possibility that some markings were removed by abrasion. However, all six rocks also sheltered a different number of adult males, females, and nymphs when reinspected after the four days, suggesting that there had been movement of almost all individuals to and from rocks. When repeated in September 2024, a total of 32 individuals across 6 rocks were originally marked. Upon re‐inspection, no marked individuals were found under or near four of the six marked rocks. One rock, under which one adult male, one adult female, and two nymphs were initially found and marked, harboured one marked adult female when re‐examined after three days. An additional two marked nymphs plus one unmarked individual were found under a nearby rock (*ca*. 30 cm from the original rock). A second of the original 6 rocks, under which four adult females, one adult male, and one nymph were originally marked, was found to harbour one marked adult female three days later.

No cockroaches were detected outside rocks during the daytime at all sites examined; however, during a short observation period commencing 9 pm on May 23rd, 2023, adults were seen moving in the open (under cover of darkness). During the 20‐min nighttime transect, 7 adult males, 10 adult females, and no nymphs were sighted.

### 3.2 Aggregation Size and Morphometrics

**FIGURE 3:**
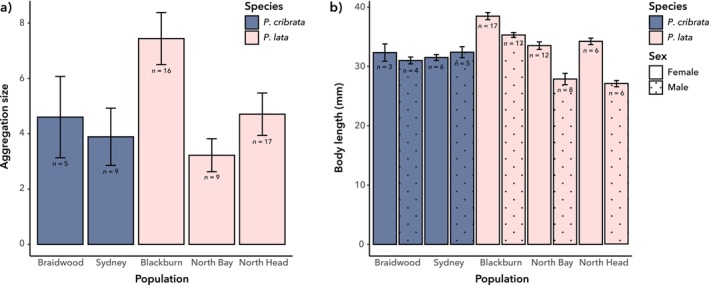
Comparisons of five populations of *P. cribrata* and *P. lata* . Bars are labelled with sample size (*n*). (a) Mean aggregation size (+/− standard error). (b) Mean body length (+/− standard error) of adult males and females.

The total number of individuals aggregating either under rocks ( *P. lata* ) or within rotten wood ( *P. cribrata* ) varied significantly between populations (*χ* ^2^_4_ = 11.746, *p* = 0.019; Figure 3a). Specifically, aggregations were larger on Blackburn Island than in North Bay (post hoc *p* = 0.019).

Body length varied significantly between populations among both females (*χ* ^2^_4_ = 30.139, *p* < 0.001; Figure 3B) and males (*χ* ^2^_4_ = 34.842, *p* < 0.001; Figure 3b). Post hoc comparisons indicated that females from Blackburn Island were longer than those from all other populations (*p* ≤ 0.015 for all), while males from Blackburn Island were longer than those from North Bay and North Head (*p* ≤ 0.001 for both).

### 3.3 Agonistic Behaviour

**FIGURE 4:**
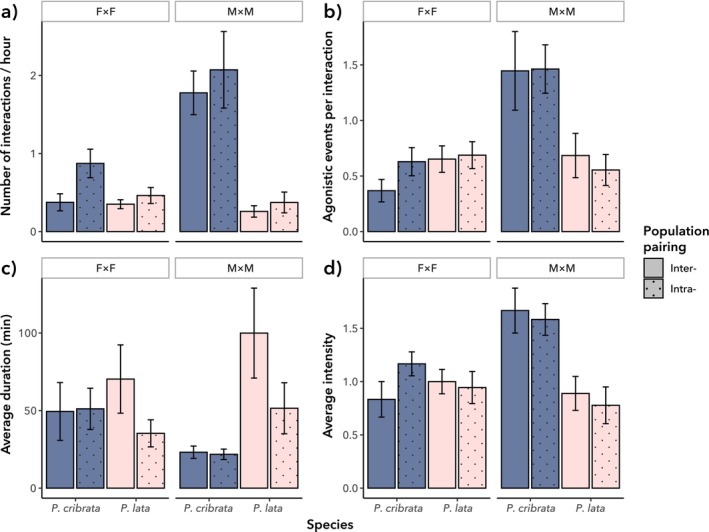
Agonistic behaviour in *P. cribrata* and *P. lata* (mean +/− standard error); F × F = female pairings, M × M = male pairings. (a) Average number of agonistic interactions per hour. (b) The proportion of the total number of interactions that were agonistic. (c) The average duration of each agonistic interaction. (d) The average intensity of each agonistic interaction.

We found no significant differences in all four measures of agonistic behaviour among female cockroaches, irrespective of species or population pairing, with no significant interactions. In contrast, male agonistic behaviour varied significantly between *P. cribrata* and *P. lata* (Figure 4). The number of agonistic interactions per hour (*t =* 3.542, *p* < 0.01), the proportion of interactions with agonism (*t* = 2.156, *p* = 0.0359), and the intensity of agonistic interactions (*t* = 2.537, *p* = 0.0143) were all significantly higher in *P. cribrata* (Figure 4a,b,d). However, the average duration of agonistic interactions was not significantly different between the species (*t* = 1.969, *p* = 0.0545; Figure 4c). None of the outcomes varied significantly based on population pairing, nor were there significant interactions between species identity and population pairing.

**FIGURE 5:**
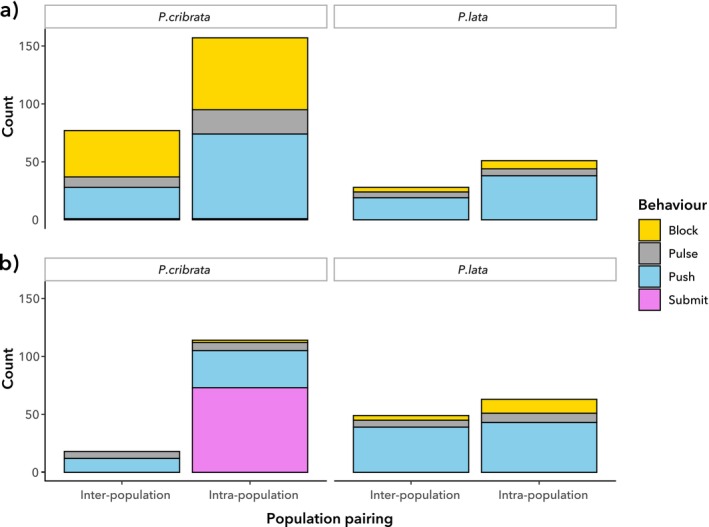
Proportions of individual behaviours in agonistic interactions. (a) Behaviours in male–male interactions. (b) Behaviours in female–female interactions.

The frequencies of different types of agonistic behaviour varied significantly between male *P. cribrata* and *P. lata* (MANOVA: *F*_1,47_ = 17.406, *p* < 0.01; Figure 5). Specifically, there was a significantly higher incidence of blocking in *P. cribrata* (*F*_1,50_ = 68.953, *p* < 0.01). Behavioural frequencies did not vary significantly between population pairings (*F*_1,47_ = 0.245, *p* = 0.911), nor was there a significant interaction between species and population pairing (*F*_1,47_ = 0.884, *p* = 0.481). For female agonistic behaviours, we found a significant interaction between species and population pairing (MANOVA: *F*_4,47_ = 8.475, *p* < 0.01; Figure 5). This was specifically driven by a significant interaction between species and population pairing for submission rate (*F*_1,50_ = 18.240, *p* < 0.01), whereby submission only occurred in intra‐population pairings of *P. cribrata* .

### 3.4 Courtship Behaviour

**FIGURE 6:**
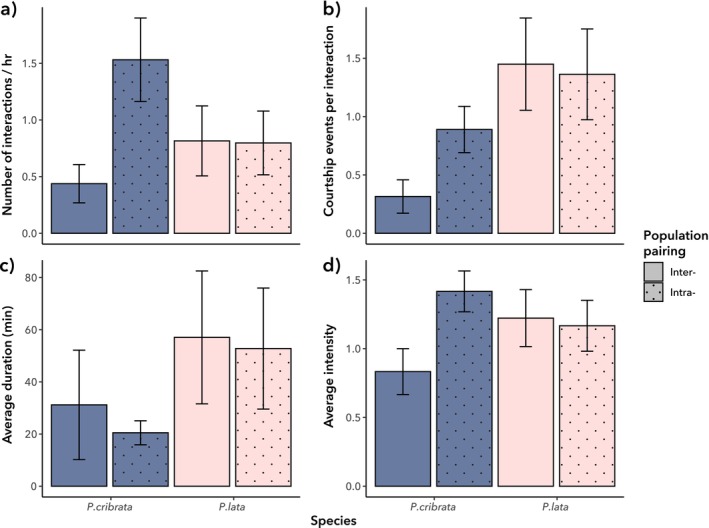
Courtship behaviour in *P. cribrata* and *P. lata* based on observation of male–female pairs (mean +/− standard error). (a) Average number of courtship interactions per hour. (b) The proportion of the total number of interactions that involved courtship. (c) The average duration of each courtship interaction. (d) The average intensity of each courtship interaction.

**FIGURE 7:**
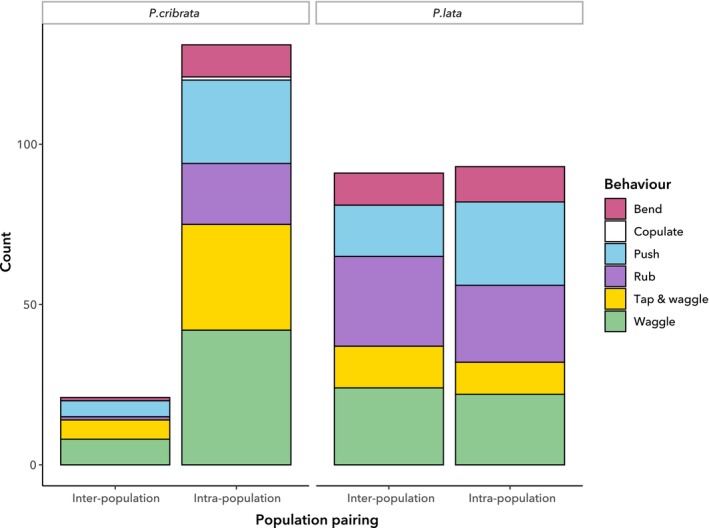
Counts of individual behaviours in courtship interactions, based on observations of male–female pairings.

None of the four measures of courtship behaviour varied significantly between species or population pairings, and we found no significant interactions between these factors (Figure 6). However, the frequencies of different courtship behaviours did vary significantly between *P. cribrata* and *P. lata* (*F*_1,45_ = 10.246, *p* < 0.01), but not based on population pairings (*F*_1,45_ = 1.032, *p* = 0.418). There was no significant interaction between these factors (*F*_1,45_ = 2.081, *p* = 0.0743). *Panesthia cribrata* was found to perform the tap and waggle behaviour significantly more frequently than *P. lata* (*F*_1,50_ = 24.370, *p* < 0.01; Figure 7).

Finally, we observed courtship behaviours in six male–male trials of *P. lata* . The behaviours were all performed by males from Blackburn Island when paired with individuals from Blackburn Island (*n* = 1 trial), North Bay (*n* = 3 trials), and North Head (*n* = 2 trials).

### 3.5 Kinship and Genetic Structure

Kinship estimates were conducted using the full dataset, consisting of both the Blackburn Island aggregations and putative unrelated individuals from Blackburn Island, North Bay, and Roach Island. Pedigree analysis with *Sequoia* did not detect any parent, sibling, or grandparent relationships among the Blackburn Island aggregations. In concordance, pairwise kinship analyses did not detect any close relatives (*k* > 0.125) within any of the aggregations. Average pairwise kinship within each aggregation was very similar to the average pairwise kinship between the remaining individuals from Blackburn Island (Table 3).

**TABLE 3:** Average pairwise kinship (excluding self‐kinship) within and between aggregations of *P. lata* on Blackburn Island.

Group	*N*	Average pairwise kinship
Aggregation 1	3	0.0650
Aggregation 2	3	0.0597
Aggregation 3	9	0.0625
Aggregation 4	9	0.0636
Separate aggregations	11	0.0636

*Note:* Values calculated in *SNPRelate*. *N* = sample size.

**FIGURE 8:**
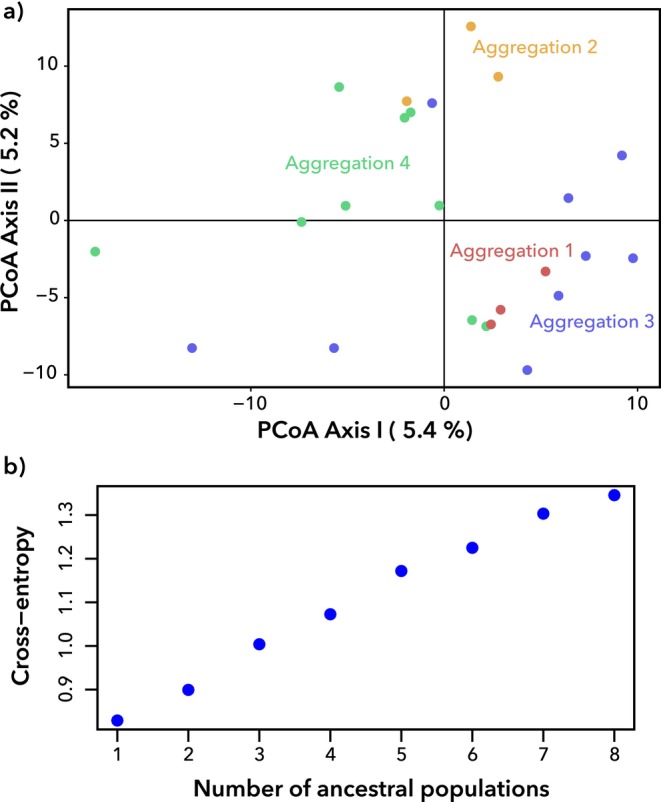
Genetic structure across four nesting aggregations of *Panesthia lata* on Blackburn Island. (a) PCoA results based on 8926 nuclear SNPs. (b) Cross‐entropy scores for *K* = 1–8 genetic clusters calculated using sNMF analysis, based on 6471 nuclear SNPs.

Analyses of genetic structure were undertaken using only the four nesting aggregations from Blackburn Island. No structure was evident based on PCoA (Figure 8a). Similarly, clustering analysis with sNMF optimised at *K* = 1, indicating an absence of genetic structure (Figure 8b). Visualisations of structure and admixture at higher *K* values did not reveal any patterns corresponding to the four aggregations (Figure S1).

## 4 Discussion

### 4.1 Behavioural Shifts Following Island Colonisation

To the best of our knowledge, this study represents the first experimental comparison of behaviour in an island invertebrate with its mainland sister species. We demonstrate that *P. lata* differs from *P. cribrata* along several ecological and behavioural axes, and that these differences are conserved among its different island populations. Most strikingly, male *P. lata* were found to engage in fewer and less intense agonistic interactions than *P. cribrata* . While the average duration of interactions was longer in *P. lata* , this appears to be caused by the lower intensity of agonism, which delayed the submission and retreat of the losing individual (S. Coady, pers. obs.). Male agonism in *Panesthia* relates to competition during dispersal and mate‐finding (Bell et al. 2007; O'Neill et al. 1987; Rugg and Rose 1984; Seelinger and Seelinger 1983), hence our findings suggest a shift in intraspecific competitive dynamics on the LHIG. Due to limitations on the collection of the critically endangered *P. lata* , we acknowledge that the sample size places bounds on interpretation. Nonetheless, the consistency of results across different behavioural metrics and different agonistic behaviours underscores their robustness.

Reduced intra‐specific aggression is a fundamental pattern in vertebrate island ecology. Such behavioural shifts have been observed across a broad range of organisms that include mammals (Adler and Levins 1994; Goltsman et al. 2005), reptiles (Pafilis et al. 2011; Pafilis et al. 2009; Raia et al. 2010), and birds (Jezierski et al. 2024). Two leading hypotheses suggest that this trend is tied to higher population densities on islands, which are linked to lower intraspecific agonism either due to greater resource availability or because the higher frequency of conspecific interactions elevates energetic and fitness costs of agonism (MacArthur et al. 1972; Stamps and Buechner 1985; Whittaker et al. 2023; Wright 1980). It is presently unclear which of these, if any, applies to *P. lata* . We find equivocal evidence of larger aggregation sizes (as a proxy for population density) in *P*. *lata* than *P. cribrata* , although the number of aggregations per unit area appears larger on the LHIG than in mainland Australia. Moreover, without comparisons across multiple generations, we cannot presently discern whether the behavioural differences represent plastic responses to differing ecological conditions or whether they represent fixed, evolved differences. Long‐term culture of *Panesthia*, coupled with granular genomic comparisons, will be necessary to untangle the genetic underpinnings.

In contrast to agonistic interactions, we detected no significant differences in the overall frequency or duration of courtship between *P. lata* and *P. cribrata* , and only slight variation in the frequencies of individual behaviours. Courtship behaviour is highly conserved across the Blattodea, which display only four mating strategies (Lizée et al. 2017; Sreng 1993). Courtship has also been overlooked in comparisons of island and mainland taxa, although reproductive studies suggest that island species produce fewer and/or smaller clutches (reviewed by Baeckens and Van Damme 2020; Novosolov and Meiri 2013). Due to the long life cycles of *P. lata* and *P. cribrata* (multiple years to reach maturity; Rugg and Rose 1990), investigation of clutch size was beyond the scope of this project.

One curious point of contrast between the species was the occurrence of male–male courtship in *P. lata* . It is unclear whether this represents a consequence of island existence, as same‐sex courtship has been documented across Blattodea (Simon and Barth 1977). Hypothesised causes include the elevation of sexual excitation, suppression of interspecific hybridisation, and maintenance of courting groups. Same‐sex behaviour can also be stimulated by breakdowns in chemical signalling, whereby males are less able to distinguish between sexually receptive and unreceptive females, leading them to court any individuals resembling a conspecific female (Dukas et al. 2006). The high rates of courtship relative to copulation observed presently suggest that male *Panesthia* attempt courtship regardless of female receptivity, which may encourage same‐sex courtship (although this was not observed in *P. cribrata* ). Comparisons of both same‐sex courtship and clutch size in *P. lata* , relative to additional mainland *Panesthia* species, present interesting directions for future study.

### 4.2 Genetic Structure of Aggregations in *P. lata*

Surprisingly, SNP analyses uniformly indicated that aggregations on Blackburn Island are composed of unrelated individuals. This sharply contrasts with other *Panesthia* species from Australia and Asia, in which aggregations are thought to primarily consist of parents and offspring (Ito and Osawa 2019, 2021; Rugg and Rose 1984). However, this result appears to complement the reduced intraspecific agonism in *P. lata* relative to *P. cribrata* . It also reflects our ecological observations, which suggest that individuals move between stones as frequently as daily. Unfortunately, we did not generate nuclear SNP data for *P. cribrata* , which would provide the most robust comparison between the two species. Nonetheless, ecological observations suggest that *P. cribrata* maintains relatively small parent‐offspring groups (albeit with the potential for some degree of dispersal; Rugg and Rose 1984), which points to a shift in aggregation behaviour following colonisation of the LHIG.

The evolutionary drivers of this divergent nesting strategy are not clear. The wooden galleries occupied by *P. cribrata* provide both food and shelter, with little benefit from movement between microhabitats. In contrast, *P. lata* forages beyond resident stones or logs (Carlile et al. 2018), which may encourage migration between family groups. We also note that stones are relatively scarce below the *Ficus* tree on Blackburn Island, which could drive unrelated individuals to share shelters. The Australian mainland species *Panesthia matthewsi* is a close relative of *P. cribrata* and *P. lata* (Adams, Walker, Rose, Jones, et al. 2025) and is known to occupy similar microhabitats to *P. lata* (shallow burrows below stones or leaf litter). A comparison of aggregation behaviour in *P. lata* and *P. matthewsi* would further illuminate whether the shift away from obligate log‐boring is consistently associated with changes in intra‐aggregation kinship, although such an approach could be confounded by the species' common ancestry. Alternatively, the apparent permeability of the aggregations in *P. lata* may be tied to inbreeding. Background levels of kinship on Blackburn Island were substantially elevated, and aggregation preferences in *Panesthia* are based in part on chemical cues that distinguish relatives from non‐relatives (Billingham et al. 2010). Thus, inbreeding in *P. lata* may interfere with the maintenance of family groups. This latter hypothesis may be examined with behavioural experiments examining association preferences between known close relatives and unrelated individuals.

The island syndrome does not circumscribe a consistent suite of adaptations present in all species, but rather a loose collection of evolutionary tendencies (Jezierski et al. 2024). In line with this perspective, our behavioural and genetic analyses reveal changes in agonism and aggregation behaviour, but not necessarily reproduction, in *P. lata* . Importantly, these changes appear linked to the unique conditions of the insular LHIG (Novosolov and Meiri 2013; Whittaker et al. 2023). Namely, the reductions in agonism and disruption of kin groups may be linked to the higher population densities and/or intensified inbreeding in *P. lata* . It is unclear why invertebrates have been previously overlooked in studies of island behaviour (Gavriilidi et al. 2022), as we demonstrate that *P. lata* is an informative model of island syndrome. Nonetheless, adaptations to islands vary greatly even in established model groups such as passerine birds and rodents (reviewed by Novosolov and Meiri 2013); thus, we emphasise the need for further research across a wide range of invertebrate groups.

### 4.3 Inter‐Population Differences and Conservation of *P. lata*

Finally, in addition to our examination of interspecific behavioural differences, we also undertook morphological and ecological comparisons between populations of *P. lata* . We identified appreciable differences in body length, whereby the North Bay and North Head individuals were smaller and displayed more pronounced sexual size dimorphism (SSD) than those on Blackburn Island. Comparisons to *P. cribrata* suggest that there have been both increases in female size on Blackburn Island and reductions in male body length on LHI. Blackburn Island and LHI have only recently been isolated (*ca*. 10–100 ka; Adams, Ewart, Carlile, Rose, et al. 2025); however, body size is relatively labile within *Panesthia* (based on morphological differences between populations on Blackburn and Roach Islands; H.A. Rose, pers. obs.).

Islands are thought to select for greater SSD, due to relaxed interspecific competition and/or intensified intraspecific competition (while this superficially contradicts our previous findings of lower individual agonism, theory predicts that the reduction in individual agonism on islands is counterbalanced by more frequent competitive interactions, such that overall intraspecific competition increases; Blanckenhorn 2005; Meiri et al. 2014). Neither factor obviously explains the divergent morphological trajectories on Blackburn Island and LHI, as our behavioural assays found no difference in agonism across populations, and habitats on both islands appear qualitatively similar (H.A. Rose, pers. obs.). An alternative possibility is that the reduction in male body size may reflect predation from black rats on LHI (e.g., Benítez‐López et al. 2021), which were historically absent on Blackburn Island. Presuming that male *P. lata* disperse more actively (as in other *Panesthia*; Rugg and Rose 1984), then males may have experienced stronger predation pressure and selection for smaller size. Museum specimens of *P. lata* collected from Blackburn Island and LHI before rodent introduction show no obvious SSD, albeit with a modest sample size (*n* < 20; M.W.D. Adams, pers. obs.).

Despite the morphological differences, we found that neither courtship nor agonistic behaviour varied between inter‐ and intra‐population assays. This is promising for the success of any future conservation translocation between islands: current guidelines recommend evidence of both genetic and ecological compatibility prior to translocation (Hedrick and Fredrickson 2010). While the present results should be further developed with captive‐crossing studies (Stringer and Chappell 2008), they nonetheless offer preliminary evidence that translocations between North Head and North Bay, or even Blackburn Island, would not disrupt the species' reproductive ecology.

## 5 Conclusions

Here we have presented the first experimental comparison of an island‐mainland pair of invertebrate species. Our findings of reduced intraspecific agonism, increased population density, and more permeable aggregations in *P. lata* align with ecological and behavioural adaptations seen in island vertebrates. On the other hand, while the reproductive ecology of *P. lata* requires more detailed examination in the future, we found no evidence of shifts in courtship behaviour relative to *P. cribrata* . Overall, we provide novel evidence that insect behaviour follows somewhat similar trajectories to vertebrates in island ecosystems.

## Author Contributions

**Susannah K. Coady:** conceptualization (equal), data curation (equal), formal analysis (equal), investigation (equal), methodology (equal), writing – original draft (equal), writing – review and editing (equal). **Maxim W. D. Adams:** data curation (equal), formal analysis (equal), writing – original draft (equal), writing – review and editing (equal). **Harley A. Rose:** conceptualization (equal), resources (equal), resources (equal), supervision (supporting), supervision (supporting), writing – review and editing (equal), writing – review and editing (equal). **James A. Walker:** data curation (equal), investigation (equal), supervision (supporting), writing – review and editing (equal). **Ian Hutton:** investigation (supporting), writing – review and editing (equal). **Ros Gloag:** conceptualization (equal), investigation (equal), methodology (equal), supervision (equal), writing – review and editing (equal). **Nathan Lo:** conceptualization (equal), data curation (equal), funding acquisition (equal), investigation (equal), supervision (equal), writing – review and editing (equal).

## Conflicts of Interest

The authors declare no conflicts of interest.

## Supporting information


**Appendix S1:** ece372402‐sup‐0001‐AppendixS1.docx.

## Data Availability

Raw data and R scripts used in this manuscript are available on Dryad: https://doi.org/10.5061/dryad.fbg79cp75.
